# Bound on optimal local discrimination of multipartite quantum states

**DOI:** 10.1038/s41598-022-18496-y

**Published:** 2022-08-19

**Authors:** Donghoon Ha, Jeong San Kim

**Affiliations:** Department of Applied Mathematics and Institute of Natural Sciences, Kyung Hee University, Yongin, 17104 Republic of Korea

**Keywords:** Quantum information, Information theory and computation

## Abstract

We consider the unambiguous discrimination of multipartite quantum states and provide an upper bound for the maximum success probability of optimal local discrimination. We also provide a necessary and sufficient condition to realize the upper bound. We further establish a necessary and sufficient condition for this upper bound to be saturated. Finally, we illustrate our results using examples in multidimensional multipartite quantum systems.

## Introduction

Quantum nonlocality is a quintessential phenomenon of multipartite quantum systems which does not have any classical counterpart. Entanglement is one of the most representative nonlocal quantum correlations which cannot be realized only by *local operations and classical communication* (LOCC)^1,2^. It is known that the nonlocal property of quantum entanglement can be used as a resource in many quantum information processing tasks^3^.

Quantum nonlocal phenomenon can also arise in multipartite quantum state discrimination, which is an essential process for efficient information transfer in quantum communication. In general, orthogonal quantum states can be discriminated with certainty, whereas such discrimination is impossible for nonorthogonal quantum states. Along this line, state discrimination strategies are needed to discriminate nonorthogonal quantum states at least with some nonzero probability^4–7^. However, some orthogonal states of multipartite quantum systems cannot be discriminated with certainty when the available measurements are limited to LOCC measurements^8^. As orthogonal states can always be discriminated with certainty when there is no limitation of possible measurement, this limited discrimination ability of LOCC measurement reveals the nonlocal phenomenon inherent in quantum state discrimination.

Nonlocal phenomenon of quantum state discrimination can also arise in discriminating nonorthogonal states of multipartite quantum systems; it is known that some nonorthogonal states cannot be optimally discriminated using only LOCC^9–11^. For this reason, much attention has been shown for the optimal local discrimination of multipartite quantum states^12–19^. Nevertheless, realizing optimal local discrimination still remains a challenging task because it is hard to have a good mathematical characterization of LOCC.

One efficient way to overcome this difficulty is to investigate possible upper bounds for the maximum success probability of optimal local discrimination. For a better understanding of optimal local discrimination, it is also important to establish good conditions realizing such upper bounds. Recently, an upper bound of maximum success probability was established in local minimum-error discrimination of bipartite quantum states. Moreover, a necessary and sufficient condition was also provided for this upper bound to be saturated^20^.

Here, we consider *unambiguous discrimination* (UD)^21–24^ among multipartite quantum states of arbitrary dimensions and provide an upper bound for the maximum success probability of optimal local discrimination. Moreover, we provide a necessary and sufficient condition to realize this upper bound. We also establish a necessary and sufficient condition for this upper bound to be saturated. Finally, we illustrate our results using examples in multidimensional multipartite quantum systems.

This paper is organized as follows. In the “Results” Section, we first recall the definition and some properties about separable operators and separable measurements in multipartite quantum systems. We further recall the definition of UD and provide some useful properties of optimal UD (Proposition 1). As the main results of this paper, we provide an upper bound for the maximum success probability of optimal local discrimination by using a certain class of Hermitian operators acting on multipartite Hilbert space (Theorem 1). Moreover, we provide a necessary and sufficient condition for the Hermitian operator to realize this upper bound (Theorem 2 and Corollary 1). We also establish a necessary and sufficient condition for this upper bound to be saturated (Corollary 2). We illustrate our results by examples in multidimensional multipartite quantum systems (Examples 1 and 2). In the “Methods” Section, we provide a detail proof of Theorem 1. In the “Discussion” Section, we summarize our results and discuss possible future works related to our results.

## Results

In multipartite quantum systems, a state is described by a density operator $\rho$ acting on a multipartite Hilbert space ${\mathscr {H}}=\bigotimes_{k=1}^{m}{\mathscr {H}}_{k}$ consisting of the subsystems ${\mathscr {H}}_{k}\cong {\mathbb {C}}^{d_{k}}$ with $m\geqslant 2$ and positive integers $d_{k}$ for $k=1,\ldots ,m$. A measurement is expressed by a *positive operator-valued measure*(POVM) $\{M_{i}\}_{i}$ that is a set of positive-semidefinite operators $M_{i}\succeq 0$ on ${\mathscr {H}}$ satisfying completeness relation $\sum _{i}M_{i}=\mathbbm {1}$, where $\mathbbm {1}$ is the identity operator on ${\mathscr {H}}$. The probability of obtaining the measurement outcome corresponding to $M_{j}$ is $\mathrm {Tr}(\rho M_{j})$ for the prepared state $\rho$.

A positive-semidefinite operator *E* (not necessarily a state) on ${\mathscr {H}}$ is called *separable* if it can be represented as a summation of positive-semidefinite product operators, that is,

1 $$\begin{aligned} \begin{array}{c} E=\sum _{l}\bigotimes _{k=1}^{m}E_{l,k}, \end{array} \end{aligned}$$

where $E_{l,k}$ is a positive-semidefinite operator on ${\mathscr {H}}_{k}$ for each $k=1,\ldots ,m$. We denote the set of all positive-semidefinite *separable* operators on ${\mathscr {H}}$ as

2 $$\begin{aligned} \mathrm {SEP}=\big \{ E\,\big |\, E\text{: } \text{ a } \text{ positive-semidefinite } \text{ separable } \text{ operator } \text{ acting } \text{ on } {\mathscr {H}}\big \}. \end{aligned}$$

A measurement $\{M_{i}\}_{i}$ is called a *LOCC measurement* if it can be implemented by LOCC, and it is called *separable* if $M_{i}\in \mathrm {SEP}$ for all *i*. It is known that every LOCC measurement is separable^1^.

A simple example of LOCC measurement is $\{M_{1,i_{1}}\otimes \cdots \otimes M_{m,i_{m}}\}_{i_{1},\ldots ,i_{m}}$ where $\{M_{l,i_{l}}\}_{i_{l}}$ is a POVM on the *l*th subsystem for each $l=1,\ldots ,m$. This separable measurement can be implemented by local measurements and classical communication; For each $l=1,\ldots ,m$, a local measurement $\{M_{l,i_{l}}\}_{i_{l}}$ is performed on the *l*th subsystem; If it is confirmed through classical communication that the measurement result of the *l*th subsystem is $M_{l,i_{l}}$ for each $l=1,\ldots ,m$, the measurement result of the whole system becomes $M_{1,i_{1}}\otimes \cdots \otimes M_{m,i_{m}}$. Thus, $\{M_{1,i_{1}}\otimes \cdots \otimes M_{m,i_{m}}\}_{i_{1},\ldots ,i_{m}}$ is a LOCC measurement.

Here, we consider the situation of discriminating *multipartite* quantum states $\rho _{1},\ldots ,\rho _{n}$ where the state $\rho _{i}$ is prepared with the probability $\eta _{i}$. We denote this situation as an ensemble $\mathscr {E}=\{\eta _{i},\rho _{i}\}_{i=1}^{n}$. Moreover, we consider the discrimination of the multipartite state ensemble $\mathscr {E}$ using a measurement $\{M_{i}\}_{i=0}^{n}$ where $M_{0}$ gives inconclusive results about the prepared state and $M_{i}$ provides conclusive results of $\rho _{i}$ for each $i=1,\ldots ,n$. For the conclusive results to be unambiguous, so-called *no-error condition* is required;

3 $$\begin{aligned} \mathrm {Tr}(\rho _{i}M_{j})=0~~\forall i,j=1,\ldots ,n~\text{ with }~i\ne j. \end{aligned}$$

By defining

4 $$\begin{aligned} \mathrm {Pos}_{i}(\mathscr {E})= \{E\succeq 0\,|\,\mathrm {Tr}(\rho _{j}E)=0~\forall j=1,\ldots ,n~\text {with}~j\ne i\}, \end{aligned}$$

Eq. (3) can be rewritten as

5 $$\begin{aligned} M_{i}\in \mathrm {Pos}_{i}(\mathscr {E})~~\forall i=1,\ldots ,n. \end{aligned}$$

We say that a measurement $\{M_{i}\}_{i=0}^{n}$ is *unambiguous* if it satisfies the no-error condition in Eq. (5).

The *optimal UD* of $\mathscr {E}$ is to minimize the probability of obtaining inconclusive results. Equivalently, the optimal UD of $\mathscr {E}$ is to achieve the optimal success probability of $\mathscr {E}=\{\eta _{i},\rho _{i}\}_{i=1}^{n}$ defined as

6 $$\begin{aligned} p_{\mathrm{G}}(\mathscr {E})=\max _{\begin{array}{c} \mathrm POVM\\ \mathrm {\rm{with}}~(5) \end{array}}\sum _{i=1}^{n}\eta _{i}\mathrm {Tr}(\rho _{i}M_{i}), \end{aligned}$$

where the maximum is taken over all possible unambiguous measurements. The following proposition provide a necessary and sufficient condition of an *optimal* unambiguous measurement realizing $p_{\mathrm{G}}(\mathscr {E})$^25^.

### Proposition 1

For given ensemble $\mathscr {E}=\{\eta _{i},\rho _{i}\}_{i=1}^{n}$, an unambiguous measurement $\{M_{i}\}_{i=0}^{n}$ provides the optimal success probability $p_{\mathrm{G}}(\mathscr {E})$ if and only if there is a Hermitian operator *K* satisfying the following condition,

7a $$\begin{aligned}&K\succeq 0, \end{aligned}$$

7b $$\begin{aligned}&\mathrm {Tr}(M_{0}K)=0, \end{aligned}$$

7c $$\begin{aligned}&K-\eta _{i}\rho _{i}\in \mathrm {Pos}_{i}^{*}(\mathscr {E})~\forall i=1,\ldots ,n, \end{aligned}$$

7d $$\begin{aligned}&\mathrm {Tr}[M_{i}(K-\eta _{i}\rho _{i})]=0~\forall i=1,\ldots ,n, \end{aligned}$$

where $\mathrm {Pos}_{i}^{*}(\mathscr {E})$($i=1,\ldots ,n$) is the dual set of $\mathrm {Pos}_{i}(\mathscr {E})$ defined as

8 $$\begin{aligned} \mathrm {Pos}_{i}^{*}(\mathscr {E})=\{ A\,|\,A^{\dagger }=A,~\mathrm {Tr}(AB)\geqslant 0~\forall B\in \mathrm {Pos}_{i}(\mathscr {E})\}. \end{aligned}$$

In this case, we have

9 $$\begin{aligned} \begin{array}{c} p_\mathrm{G}(\mathscr {E})=\sum \limits _{i=1}^{n}\eta _{i}\mathrm {Tr}(\rho _{i}M_{i})=\mathrm {Tr}K. \end{array} \end{aligned}$$

For each $\mathrm {Pos}_{i}(\mathscr {E})$ in Eq. (4), we denote the subset of separable operators,

10 $$\begin{aligned} \mathrm {SEP}_{i}(\mathscr {E})=\mathrm {Pos}_{i}(\mathscr {E})\cap \mathrm {SEP}= \{E\in \mathrm {SEP}\,|\,\mathrm {Tr}(\rho _{j}E)=0~\forall j=1,\ldots ,n~\text {with}~j\ne i\}. \end{aligned}$$

The unambiguous measurement $\{M_{i}\}_{i=0}^{n}$ is called separable if

11 $$\begin{aligned} M_{0}\in \mathrm {SEP},~M_{i}\in \mathrm {SEP}_{i}(\mathscr {E})~\forall i=1,\ldots ,n, \end{aligned}$$

where $\mathrm {SEP}$ is defined in Eq. (2).

When the available measurements are limited to separable unambiguous measurements, we denote the maximum success probability by

12 $$\begin{aligned} p_{\mathrm{SEP}}(\mathscr {E})=\max _{\begin{array}{c} \mathrm POVM\\ \mathrm {\rm{with}}~(11) \end{array}}\sum _{i=1}^{n}\eta _{i}\mathrm {Tr}(\rho _{i}M_{i}). \end{aligned}$$

We use $p_{\mathrm{L}}(\mathscr {E})$ to denote the maximum of success probability that can be obtained using LOCC unambiguous measurements, that is,

13 $$\begin{aligned} p_{\mathrm{L}}(\mathscr {E})=\max _{\begin{array}{c} \mathrm LOCC~POVM\\ \mathrm {\rm{with}}~(5) \end{array}}\sum _{i=1}^{n}\eta _{i}\mathrm {Tr}(\rho _{i}M_{i}). \end{aligned}$$

For a given ensemble $\mathscr {E}=\{\eta _{i},\rho _{i}\}_{i=1}^{n}$, we define $q_{\mathrm{SEP}}(\mathscr {E})$ as the minimum quantity

14 $$\begin{aligned} q_{\mathrm{SEP}}(\mathscr {E})=\min \mathrm {Tr}H \end{aligned}$$

over all possible Hermitian operator *H* satisfying

15a $$\begin{aligned}&H\in \mathrm {SEP}^{*}, \end{aligned}$$

15b $$\begin{aligned}&H-\eta _{i}\rho _{i}\in \mathrm {SEP}_{i}^{*}(\mathscr {E})~\forall i=1,\ldots ,n, \end{aligned}$$

where $\mathrm {SEP}^{*}$ and $\mathrm {SEP}_{i}^{*}(\mathscr {E})$($i=1,\ldots ,n$) are the dual sets of $\mathrm {SEP}$ and $\mathrm {SEP}_{i}(\mathscr {E})$ in Eqs. (2) and (10), respectively, that is,

16 $$\begin{aligned} \begin{array}{rcl} \mathrm {SEP}^{*}&{}=&{}\{ A\,|\,A^{\dagger }=A,~\mathrm {Tr}(AB)\geqslant 0~\forall B\in \mathrm {SEP}\},\\ \mathrm {SEP}_{i}^{*}(\mathscr {E})&{}=&{}\{ A\,|\,A^{\dagger }=A,~\mathrm {Tr}(AB)\geqslant 0~\forall B\in \mathrm {SEP}_{i}(\mathscr {E})\}. \end{array} \end{aligned}$$

Note that $\mathrm {SEP}^{*}$ contains all positive-semidefinite operators because every element of $\mathrm {SEP}$ is positive semidefinite. Due to the similar reason, $\mathrm {SEP}_{i}^{*}(\mathscr {E})$($i=1,\ldots ,n$) contains all positive-semidefinite operators.

The following theorem shows that $q_{\mathrm{SEP}}(\mathscr {E})$ is equal to $p_{\mathrm{SEP}}(\mathscr {E})$ in Eq. (12). The proof of Theorem 1 is provided in the “Methods” Section.

### Theorem 1

For a multipartite quantum state ensemble $\mathscr {E}=\{\eta _{i},\rho _{i}\}_{i=1}^{n}$,

17 $$\begin{aligned} p_{\mathrm{SEP}}(\mathscr {E})=q_{\mathrm{SEP}}(\mathscr {E}). \end{aligned}$$

For a given ensemble $\mathscr {E}=\{\eta _{i},\rho _{i}\}_{i=1}^{n}$, the following theorem provides a necessary and sufficient condition on a Hermitian operator *H* to realize $q_{\mathrm{SEP}}(\mathscr {E})$.

### Theorem 2

For a multipartite quantum state ensemble $\mathscr {E}=\{\eta _{i},\rho _{i}\}_{i=1}^{n}$, a Hermitian operator *H* satisfying Condition (15) gives $q_\mathrm{SEP}(\mathscr {E})$ if and only if there is a separable unambiguous measurement $\{M_{i}\}_{i=0}^{n}$ such that,

18a $$\begin{aligned} \mathrm {Tr}(M_{0}H)=0, \end{aligned}$$

18b $$\begin{aligned} \mathrm {Tr}[M_{i}(H-\eta _{i}\rho _{i})]=0~~\forall i=1,\ldots ,n. \end{aligned}$$

In this case, we have

19 $$\begin{aligned} \begin{array}{c} q_\mathrm{SEP}(\mathscr {E})=\mathrm {Tr}H=\sum \limits _{i=1}^{n}\eta _{i}\mathrm {Tr}(\rho _{i}M_{i}). \end{array} \end{aligned}$$

### Proof

For the necessity, suppose that *H* is a Hermitian operator providing $q_{\mathrm{SEP}}(\mathscr {E})$. We also denote $\{M_{i}\}_{i=0}^{n}$ as a separable unambiguous measurement giving $p_{\mathrm{SEP}}(\mathscr {E})$. From Conditions (11) and (15), we have

20 $$\begin{aligned} \mathrm {Tr}(M_{0}H)\geqslant 0,~\mathrm {Tr}[M_{i}(H-\eta _{i}\rho _{i})]\geqslant 0~\forall i=1,\ldots ,n. \end{aligned}$$

We also note that

21 $$\begin{aligned} \begin{array}{c} \mathrm {Tr}(M_{0}H)+\sum \limits _{i=1}^{n}\mathrm {Tr}[M_{i}(H-\eta _{i}\rho _{i})] =\mathrm {Tr}H-\sum \limits _{i=1}^{n}\eta _{i}\mathrm {Tr}(\rho _{i}M_{i}) =q_{\mathrm{SEP}}(\mathscr {E})-p_{\mathrm{SEP}}(\mathscr {E})=0, \end{array} \end{aligned}$$

where the first equality follows from $\sum _{i=0}^{n}M_{i}=\mathbbm {1}$, the second equality is due to the assumption of *H* and $\{M_{i}\}_{i=0}^{n}$, and the last equality is by Theorem 1. Inequality (20) and Eq. (21) lead us to Condition (18). Therefore, $\{M_{i}\}_{i=0}^{n}$ is a separable unambiguous measurement satisfying Condition (18).

For the sufficiency, we assume that $\{M_{i}\}_{i=0}^{n}$ is a separable unambiguous measurement and *H* is a Hermitian operator satisfying Conditions (15) and (18). This assumption implies,

22 $$\begin{aligned} \begin{array}{c} q_{\mathrm{SEP}}(\mathscr {E}) =p_{\mathrm{SEP}}(\mathscr {E}) \geqslant \sum \limits _{i=1}^{n}\eta _{i}\mathrm {Tr}(\rho _{i}M_{i}) =\sum \limits _{i=1}^{n}\eta _{i}\mathrm {Tr}(\rho _{i}M_{i}) +\mathrm {Tr}(M_{0}H)+\sum \limits _{i=1}^{n}\mathrm {Tr}[M_{i}(H-\eta _{i}\rho _{i})] =\mathrm {Tr}H \geqslant q_{\mathrm{SEP}}(\mathscr {E}), \end{array} \end{aligned}$$

where the first equality follows from Theorem 1, the second equality is from Condition (18), the last equality is due to $\sum _{i=0}^{n}M_{i}=\mathbbm {1}$, and the first and second inequalities are from the definitions of $p_\mathrm{SEP}(\mathscr {E})$ and $q_{\mathrm{SEP}}(\mathscr {E})$, respectively. Inequality (22) leads us to $\mathrm {Tr}H=q_\mathrm{SEP}(\mathscr {E})$. Therefore, *H* is a Hermitian operator giving $q_{\mathrm{SEP}}(\mathscr {E})$. $\square$

From Theorems 1 and 2, we have the following corollary providing a necessary and sufficient condition on a separable unambiguous measurement $\{M_{i}\}_{i=0}^{n}$ to realize $p_{\mathrm{SEP}}(\mathscr {E})$.

### Corollary 1.

For a multipartite quantum state ensemble $\mathscr {E}=\{\eta _{i},\rho _{i}\}_{i=1}^{n}$, a separable unambiguous measurement $\{M_{i}\}_{i=0}^{n}$ gives $p_\mathrm{SEP}(\mathscr {E})$ if and only if there is a Hermitian operator *H* satisfying Conditions (15) and (18). In this case, we have,

23 $$\begin{aligned} \begin{array}{c} p_\mathrm{SEP}(\mathscr {E})=\sum \limits _{i=1}^{n}\eta _{i}\mathrm {Tr}(\rho _{i}M_{i})=\mathrm {Tr}H. \end{array} \end{aligned}$$

Moreover, we have the following corollary that provides the relative ordering between $p_{\mathrm{L}}(\mathscr {E})$ and $q_\mathrm{SEP}(\mathscr {E})$.

### Corollary 2.

For a multipartite quantum state ensemble $\mathscr {E}=\{\eta _{i},\rho _{i}\}_{i=1}^{n}$,

24 $$\begin{aligned} p_{\mathrm{L}}(\mathscr {E})\leqslant q_{\mathrm{SEP}}(\mathscr {E}), \end{aligned}$$

where the equality holds if and only if there is a LOCC unambiguous measurement $\{M_{i}\}_{i=0}^{n}$ and a Hermitian operator *H* satisfying Conditions (15) and (18).

### Proof

Since every LOCC measurement is separable, $p_\mathrm{L}(\mathscr {E})\leqslant p_{\mathrm{SEP}}(\mathscr {E})$. Moreover, $p_{\mathrm{L}}(\mathscr {E})=p_{\mathrm{SEP}}(\mathscr {E})$ if and only if there is a LOCC unambiguous measurement realizing $p_\mathrm{SEP}(\mathscr {E})$. Thus, we can show from Theorem 1 and Corollary 1 that our statement is true. $\square$

Here, we provide the following example of two-qubit state ensemble $\mathscr {E}$ to illustrate how our results can be used to obtain $p_{\mathrm{G}}(\mathscr {E})$, $q_{\mathrm{SEP}}(\mathscr {E})$, and $p_{\mathrm{L}}(\mathscr {E})$.

### Example 1.

Let us consider the two-qubit state ensemble $\mathscr {E}=\{\eta _{i},\rho _{i}\}_{i=1}^{3}$ consisting of three product states with equal prior probabilities,

25 $$\begin{aligned} \begin{array}{lll} \eta _{1}=\frac{1}{3},~~\rho _{1}=|0\rangle \!\langle 0|\otimes |0\rangle \!\langle 0|,&{}\\ \eta _{2}=\frac{1}{3},~~\rho _{2}=|\nu _{+}\rangle \!\langle \nu _{+}|\otimes |\nu _{+}\rangle \!\langle \nu _{+}|,~~ |\nu _{+}\rangle =\frac{1}{2}|0\rangle +\frac{\sqrt{3}}{2}|1\rangle ,\\ \eta _{3}=\frac{1}{3},~~\rho _{3}=|\nu _{-}\rangle \!\langle \nu _{-}|\otimes |\nu _{-}\rangle \!\langle \nu _{-}|,~~|\nu _{-}\rangle =\frac{1}{2}|0\rangle -\frac{\sqrt{3}}{2}|1\rangle . \end{array} \end{aligned}$$

To obtain $p_{\mathrm{G}}(\mathscr {E})$, we use the unambiguous measurement $\{M_{i}\}_{i=0}^{3}$ with

26 $$\begin{aligned} \begin{array}{ll} M_{0}=\frac{1}{2}|\Phi _{+}\rangle \!\langle \Phi _{+}|+|\Psi _{-}\rangle \!\langle \Psi _{-}|,\\ M_{1}=\frac{5}{6}|\Phi _{1}\rangle \!\langle \Phi _{1}|,~|\Phi _{1}\rangle =\frac{3}{\sqrt{10}}|00\rangle -\frac{1}{\sqrt{10}}|11\rangle ,\\ M_{2}=\frac{5}{6}|\Phi _{2}\rangle \!\langle \Phi _{2}|,~|\Phi _{2}\rangle =\sqrt{\frac{3}{10}}|01\rangle +\sqrt{\frac{3}{10}}|10\rangle +\frac{2}{\sqrt{10}}|11\rangle ,\\ M_{3}=\frac{5}{6}|\Phi _{3}\rangle \!\langle \Phi _{3}|,~|\Phi _{3}\rangle =\sqrt{\frac{3}{10}}|01\rangle +\sqrt{\frac{3}{10}}|10\rangle -\frac{2}{\sqrt{10}}|11\rangle , \end{array} \end{aligned}$$

and Hermitian operator

27 $$\begin{aligned} \begin{array}{c} K=\frac{3}{8}|\Phi _{-}\rangle \!\langle \Phi _{-}|+\frac{3}{8}|\Psi _{+}\rangle \!\langle \Psi _{+}|, \end{array} \end{aligned}$$

where

28 $$\begin{aligned} \begin{array}{c} |\Phi _{\pm }\rangle =\frac{1}{\sqrt{2}}|00\rangle \pm \frac{1}{\sqrt{2}}|11\rangle ,~ |\Psi _{\pm }\rangle =\frac{1}{\sqrt{2}}|01\rangle \pm \frac{1}{\sqrt{2}}|10\rangle \end{array} \end{aligned}$$

are the Bell states in two-qubit systems. We will show the unambiguous measurement $\{M_{i}\}_{i=0}^{3}$ and the Hermitian operator *K* satisfy the conditions of Proposition 1 for the ensemble $\mathscr {E}=\{\eta _{i},\rho _{i}\}_{i=1}^{3}$ in Eq. (25).

For *K* of Eq. (27), Condition (7a) is obvious and Condition (7b) is also true due to the orthogonality of Bell states. For Condition (7c), we first note $|\Psi _{-}\rangle \!\langle \Psi _{-}|$ is orthogonal to each $\rho _{i}$, that is, $\mathrm {Tr}(|\Psi _{-}\rangle \!\langle \Psi _{-}|\rho _{i})=0$, $i=1,2,3$. Moreover, $|\Phi _{i}\rangle \!\langle \Phi _{i}|$ is orthogonal to $\rho _{j}$ for all $i,j=1,2,3$ with $i\ne j$. Thus, we have

29 $$\begin{aligned} \mathrm {Pos}_{i}(\mathscr {E})=\{E\succeq 0\,|\, \text{ E } \text{ acting } \text{ on } \text{ the } \text{ subspace } \text{ spanned } \text{ by } |\Phi _{i}\rangle \text{ and } |\Psi _{-}\rangle \}~~\forall i=1,2,3, \end{aligned}$$

for the ensemble $\mathscr {E}$ of Eq. (25). Now, a straightforward calculation leads us to

30 $$\begin{aligned} \langle \Phi _{i}|(K-\eta _{i}\rho _{i})|\Phi _{i}\rangle =\langle \Phi _{i}|(K-\eta _{i}\rho _{i})|\Psi _{-}\rangle =\langle \Psi _{-}|(K-\eta _{i}\rho _{i})|\Psi _{-}\rangle =0~~\forall i=1,2,3, \end{aligned}$$

and this implies

31 $$\begin{aligned} \mathrm {Tr}[(K-\eta _{i}\rho _{i})E]=0 \end{aligned}$$

for all *E* in $\mathrm {Pos}_{i}(\mathscr {E})$. From the definition of $\mathrm {Pos}_{i}^{*}(\mathscr {E})$ in Eq. (8), we have $K-\eta _{i}\rho _{i}\in \mathrm {Pos}_{i}^{*}(\mathscr {E})$ for each $i=1,2,3$, and this shows the validity of Condition (7c). Finally, Condition (7d) naturally follows from Eqs. (26) and (30). From Proposition 1, the optimal success probability $p_\mathrm{G}(\mathscr {E})$ in Eq. (6) is

32 $$\begin{aligned} \begin{array}{c} p_\mathrm{G}(\mathscr {E})=\sum \limits _{i=1}^{3}\eta _{i}\mathrm {Tr}(\rho _{i}M_{i})=\mathrm {Tr}K=\frac{3}{4}. \end{array} \end{aligned}$$

To obtain $q_{\mathrm{SEP}}(\mathscr {E})$ in Eq. (14), let us consider the Hermitian operator

33 $$\begin{aligned} \begin{array}{l} H=\frac{1}{2}|\Psi _{-}\rangle \!\langle \Psi _{-}|, \end{array} \end{aligned}$$

and the separable unambiguous measurement $\{M_{i}\}_{i=0}^{3}$ consisting of

34 $$\begin{aligned} \begin{array}{l} M_{0}=\frac{4}{9}|1\rangle \!\langle 1|\otimes |1\rangle \!\langle 1|+\frac{4}{9}|\mu _{+}\rangle \!\langle \mu _{+}|\otimes |\mu _{+}\rangle \!\langle \mu _{+}|+\frac{4}{9}|\mu _{-}\rangle \!\langle \mu _{-}|\otimes |\mu _{-}\rangle \!\langle \mu _{-}|,\\M_{1}=\frac{4}{9}|\mu _{+}\rangle \!\langle \mu _{+}|\otimes |\mu _{-}\rangle \!\langle \mu _{-}|+\frac{4}{9}|\mu _{-}\rangle \!\langle \mu _{-}|\otimes |\mu _{+}\rangle \!\langle \mu _{+}|,\\M_{2}=\frac{4}{9}|\mu _{+}\rangle \!\langle \mu _{+}|\otimes |1\rangle \!\langle 1|+\frac{4}{9} |1\rangle \!\langle 1|\otimes |\mu _{+}\rangle \!\langle \mu _{+}|,\\M_{3}=\frac{4}{9}|\mu _{-}\rangle \!\langle \mu _{-}|\otimes |1\rangle \!\langle 1|+\frac{4}{9} |1\rangle \!\langle 1|\otimes |\mu _{-}\rangle \!\langle \mu _{-}|, \end{array} \end{aligned}$$

where

35 $$\begin{aligned} \begin{array}{l} |\mu _{\pm }\rangle =\frac{\sqrt{3}}{2}|0\rangle \pm \frac{1}{2}|1\rangle . \end{array} \end{aligned}$$

We will show the Hermitian operator *H* and the separable unambiguous measurement $\{M_{i}\}_{i=0}^{3}$ satisfy the conditions of Theorem 2 for the ensemble $\mathscr {E}=\{\eta _{i},\rho _{i}\}_{i=1}^{3}$ in Eq. (25).

For *H* of Eq. (33), Condition (15a) holds due to the argument after Eq. (16) and Condition (18a) is also true from the fact that $|\Psi _{-}\rangle$ is orthogonal to $|1\rangle \otimes |1\rangle$,$|\mu _{+}\rangle \otimes |\mu _{+}\rangle$,$|\mu _{-}\rangle \otimes |\mu _{-}\rangle$. For Condition (15b), we first note every positive-semidefinite product operator orthogonal to $\rho _{2}$ and $\rho _{3}$ is proportional to $|\mu _{+}\rangle \!\langle \mu _{+}|\otimes |\mu _{-}\rangle \!\langle \mu _{-}|$ or $|\mu _{-}\rangle \!\langle \mu _{-}|\otimes |\mu _{+}\rangle \!\langle \mu _{+}|$. Moreover, every positive-semidefinite product operator orthogonal to $\rho _{1}$ and $\rho _{3(2)}$ is proportional to $|\mu _{+(-)}\rangle \!\langle \mu _{+(-)}|\otimes |1\rangle \!\langle 1|$ or $|1\rangle \!\langle 1|\otimes |\mu _{+(-)}\rangle \!\langle \mu _{+(-)}|$. Thus, we have

36 $$\begin{aligned} \begin{array}{l} \mathrm {SEP}_{1}(\mathscr {E})=\{ a|\mu _{+}\rangle \!\langle \mu _{+}|\otimes |\mu _{-}\rangle \!\langle \mu _{-}| +b|\mu _{-}\rangle \!\langle \mu _{-}|\otimes |\mu _{+}\rangle \!\langle \mu _{+}|~|~a,b\geqslant 0\},\\ \mathrm {SEP}_{2}(\mathscr {E})=\{ a|\mu _{+}\rangle \!\langle \mu _{+}|\otimes |1\rangle \!\langle 1| +b|1\rangle \!\langle 1|\otimes |\mu _{+}\rangle \!\langle \mu _{+}|~|~a,b\geqslant 0\}, \\ \mathrm {SEP}_{3}(\mathscr {E})=\{ a|\mu _{-}\rangle \!\langle \mu _{-}|\otimes |1\rangle \!\langle 1| +b|1\rangle \!\langle 1|\otimes |\mu _{-}\rangle \!\langle \mu _{-}|~|~a,b\geqslant 0\}, \end{array} \end{aligned}$$

for the ensemble $\mathscr {E}$ of Eq. (25). Now, a straightforward calculation leads us to

37 $$\begin{aligned} \begin{array}{l} \langle v|(H-\eta _{1}\rho _{1})|v\rangle =0 ~~\forall |v\rangle = |\mu _{+}\rangle \otimes |\mu _{-}\rangle , |\mu _{-}\rangle \otimes |\mu _{+}\rangle ,\\ \langle v|(H-\eta _{2}\rho _{2})|v\rangle =0 ~~\forall |v\rangle = |\mu _{+}\rangle \otimes |1\rangle , |1\rangle \otimes |\mu _{+}\rangle , \\ \langle v|(H-\eta _{3}\rho _{3})|v\rangle =0 ~~\forall |v\rangle = |\mu _{-}\rangle \otimes |1\rangle , |1\rangle \otimes |\mu _{-}\rangle , \end{array} \end{aligned}$$

and this implies

38 $$\begin{aligned} \mathrm {Tr}[(H-\eta _{i}\rho _{i})E]=0 \end{aligned}$$

for all *E* in $\mathrm {SEP}_{i}(\mathscr {E})$($i=1,2,3$). From the definition of $\mathrm {SEP}_{i}^{*}(\mathscr {E})$($i=1,2,3$) in Eq. (16), we have $H-\eta _{i}\rho _{i}\in \mathrm {SEP}_{i}^{*}(\mathscr {E})$ for each $i=1,2,3$, and this shows the validity of Condition (15b). Finally, Condition (18b) naturally follows from Eqs. (34) and (37). From Theorem 2, the minimum quantity $q_{\mathrm{SEP}}(\mathscr {E})$ in Eq. (14) is

39 $$\begin{aligned} \begin{array}{c} q_\mathrm{SEP}(\mathscr {E})=\mathrm {Tr}H=\sum \limits _{i=1}^{3}\eta _{i}\mathrm {Tr}(\rho _{i}M_{i})=\frac{1}{2}. \end{array} \end{aligned}$$

We also note that the POVM $\{M_{i}\}_{i=0}^{3}$ with Eq. (34) is a LOCC measurement because it can be implemented by performing the same local measurement $\{\frac{2}{3}|1\rangle \langle 1|,\frac{2}{3}|\mu _{+}\rangle \!\langle \mu _{+}|,\frac{2}{3}|\mu _{-}\rangle \!\langle \mu _{-}|\}$ on two subsystems. Thus, Corollary 2 and Eq. (39) lead us to

40 $$\begin{aligned} \begin{array}{c} p_{\mathrm{L}}(\mathscr {E})=q_{\mathrm{SEP}}(\mathscr {E})=\frac{1}{2}. \end{array} \end{aligned}$$

Now, we provide another example of mixed-state ensemble in multipartite high-dimensional quantum systems to illustrate the application of our results in obtaining $p_{\mathrm{G}}(\mathscr {E})$, $q_{\mathrm{SEP}}(\mathscr {E})$, and $p_{\mathrm{L}}(\mathscr {E})$.

### Example 2

For any integer $d\geqslant 3$, let us consider the following ($d-1$)-qu*d*it state ensemble $\mathscr {E}=\{\eta _{i},\rho _{i}\}_{i=1}^{d}$ consisting of *d* mixed states with equal prior probabilities,

41 $$\begin{aligned} \eta _{i}= & {} \frac{1}{d},~\rho _{i}= \frac{1}{2(d-1)}\Bigg [\mathbbm {1}-\sum _{\begin{array}{c} j=1\\ j\ne i \end{array}}^{d}(|\Lambda _{j}\rangle \!\langle \Lambda _{j}|+|\Omega _{j}\rangle \!\langle \Omega _{j}|)\Bigg ], ~i=1,\ldots ,d,\\&|\Lambda _{j}\rangle =|j-1\rangle ^{\otimes (d-1)},~ |\Omega _{j}\rangle =\frac{1}{\sqrt{d-1}}\sum _{\begin{array}{c} k=0\\ k\ne j-1 \end{array}}^{d-1} \bigotimes _{\begin{array}{c} l=0\\ k\oplus l\ne j-1 \end{array}}^{d-1}|k\oplus l\rangle , ~~j=1,\ldots ,d,\nonumber \end{aligned}$$

where $\oplus$ denotes modulo-*d* addition. To obtain $p_\mathrm{G}(\mathscr {E})$, we use the unambiguous measurement $\{M_{i}\}_{i=0}^{d}$ with

42 $$\begin{aligned} \begin{array}{c} M_{0}=\mathbbm {1}-\sum \limits _{j=1}^{d}(|\Lambda _{j}\rangle \!\langle \Lambda _{j}|+ |\Omega _{j}\rangle \!\langle \Omega _{j}|),~ M_{i}=|\Lambda _{i}\rangle \!\langle \Lambda _{i}|+ |\Omega _{i}\rangle \!\langle \Omega _{i}|,~i=1,\ldots ,d, \end{array} \end{aligned}$$

and Hermitian operator

43 $$\begin{aligned} \begin{array}{c} K=\frac{1}{2d(d-1)}\sum \limits _{j=1}^{d}(|\Lambda _{j}\rangle \!\langle \Lambda _{j}|+ |\Omega _{j}\rangle \!\langle \Omega _{j}|). \end{array} \end{aligned}$$

We will show the unambiguous measurement $\{M_{i}\}_{i=0}^{d}$ and the Hermitian operator *K* satisfy the conditions of Proposition 1 for the ensemble $\mathscr {E}=\{\eta _{i},\rho _{i}\}_{i=1}^{d}$ in Eq. (41).

For *K* of Eq. (43), Condition (7a) is obvious and Condition (7b) is also true due to the orthogonality of $\{|\Lambda _{i}\rangle ,|\Omega _{i}\rangle \}_{i=1}^{d}$. For Condition (7c), we first note both $|\Lambda _{i}\rangle \!\langle \Lambda _{i}|$ and $|\Omega _{i}\rangle \!\langle \Omega _{i}|$ are orthogonal to $\rho _{j}$, that is, $\mathrm {Tr}(|\Lambda _{i}\rangle \!\langle \Lambda _{i}|\rho _{j})=\mathrm {Tr}(|\Omega _{i}\rangle \!\langle \Omega _{i}|\rho _{j})=0$, for all $i,j=1,\ldots ,d$ with $i\ne j$.

Let $O_{1}$ be the set of all positive semidefinite operators orthogonal to $\rho _{1}$. From the definition of $\rho _{i}$ in Eq. (41), we have

44 $$\begin{aligned} O_{1}=\{E\succeq 0\,|\, E \text{ acting } \text{ on } \text{ the } \text{ subspace } \text{ spanned } \text{ by } |\Lambda _{2}\rangle ,\ldots ,|\Lambda _{d}\rangle \text{ and } |\Omega _{2}\rangle ,\ldots ,|\Omega _{d}\rangle \}. \end{aligned}$$

Now, let $O_{j}$ be the set of all positive semidefinite operators orthogonal to $\rho _{j}$ for each $j=2,\ldots ,d$. Similarly, we have

45 $$\begin{aligned} \begin{array}{l} O_{j}=\{E\succeq 0\,|\, E \text{ acting } \text{ on } \text{ the } \text{ subspace } \text{ spanned } \text{ by } |\Lambda _{1}\rangle ,\ldots ,\widehat{|\Lambda _{j}\rangle },\ldots ,|\Lambda _{d}\rangle \text{ and } |\Omega _{1}\rangle ,\ldots ,\widehat{|\Omega _{j}\rangle },\ldots ,|\Omega _{d}\rangle \}, \end{array} \end{aligned}$$

where

46 $$\begin{aligned} \begin{array}{l} \{|\Lambda _{1}\rangle ,\ldots ,\widehat{|\Lambda _{j}\rangle },\ldots ,|\Lambda _{d}\rangle \} =\{ |\Lambda _{1}\rangle ,\ldots ,|\Lambda _{j-1}\rangle ,|\Lambda _{j+1}\rangle ,\ldots ,|\Lambda _{d}\rangle \},\\ \{|\Omega _{1}\rangle ,\ldots ,\widehat{|\Omega _{j}\rangle },\ldots ,|\Omega _{d}\rangle \} = \{|\Omega _{1}\rangle ,\ldots ,|\Omega _{j-1}\rangle ,|\Omega _{j+1}\rangle ,\ldots ,|\Omega _{d}\rangle \}. \end{array} \end{aligned}$$

From the definition of $\mathrm {Pos}_{i}(\mathscr {E})$ in Eq. (4), we have

47 $$\begin{aligned} \begin{array}{c} \mathrm {Pos}_{i}(\mathscr {E}) =\bigcap _{\begin{array}{c} j=1\\ j\ne i \end{array}}^{d}O_{j} =\{E\succeq 0\,|\, E \text{ acting } \text{ on } \text{ the } \text{ subspace } \text{ spanned } \text{ by } |\Lambda _{i}\rangle \text{ and } |\Omega _{i}\rangle \}~~\forall i=1,\ldots ,d, \end{array} \end{aligned}$$

for the ensemble $\mathscr {E}$ of Eq. (41). Moreover, a straightforward calculation leads us to

48 $$\begin{aligned} \langle \Lambda _{i}|(K-\eta _{i}\rho _{i})|\Lambda _{i}\rangle =\langle \Lambda _{i}|(K-\eta _{i}\rho _{i})|\Omega _{i}\rangle =\langle \Omega _{i}|(K-\eta _{i}\rho _{i})|\Omega _{i}\rangle =0 ~\forall i=1,\ldots ,d. \end{aligned}$$

This implies Eq. (31) for all *E* in $\mathrm {Pos}_{i}(\mathscr {E})$($i=1,\ldots ,d$). From the definition of $\mathrm {Pos}_{i}^{*}(\mathscr {E})$ in Eq. (8), we have $K-\eta _{i}\rho _{i}\in \mathrm {Pos}_{i}^{*}(\mathscr {E})$ for each $i=1,\ldots ,d$, and this shows the validity of Condition (7c). Finally, Condition (7d) naturally follows from Eqs. (42) and (48). Therefore, the optimal success probability $p_{\mathrm{G}}(\mathscr {E})$ in Eq. (6) is

49 $$\begin{aligned} \begin{array}{c} p_\mathrm{G}(\mathscr {E})=\sum \limits _{i=1}^{d}\eta _{i}\mathrm {Tr}(\rho _{i}M_{i})=\mathrm {Tr}K=\frac{1}{d-1}. \end{array} \end{aligned}$$

To obtain $q_{\mathrm{SEP}}(\mathscr {E})$ in Eq. (14), let us consider the Hermitian operator

50 $$\begin{aligned} \begin{array}{c} H=\frac{1}{2d(d-1)}\sum \limits _{j=1}^{d}|\Lambda _{j}\rangle \!\langle \Lambda _{j}|, \end{array} \end{aligned}$$

and the separable unambiguous measurement $\{M_{i}\}_{i=0}^{d}$ consisting of

51 $$\begin{aligned} \begin{array}{c} M_{0}=\mathbbm {1}-\sum \limits _{j=1}^{d}|\Lambda _{j}\rangle \!\langle \Lambda _{j}|,~ M_{i}=|\Lambda _{i}\rangle \!\langle \Lambda _{i}|,~i=1,\ldots ,d, \end{array} \end{aligned}$$

where $\{|\Lambda _{j}\rangle \}_{j=1}^{d}$ is defined in Eq. (41). We will show the Hermitian operator *H* and the separable unambiguous measurement $\{M_{i}\}_{i=0}^{d}$ satisfy the conditions of Theorem 2 for the ensemble $\mathscr {E}=\{\eta _{i},\rho _{i}\}_{i=1}^{d}$ in Eq. (41).

For *H* of Eq. (50), Condition (15a) is satisfied from the argument after Eq. (16) and Condition (18a) is also true due to the orthogonality of $\{|\Lambda _{i}\rangle \}_{i=1}^{d}$. For Condition (15b), we first note that $|\Lambda _{i}\rangle \!\langle \Lambda _{i}|$ is obviously separable for all $i=1,\ldots ,d$. However, $|\Omega _{i}\rangle \!\langle \Omega _{i}|$ is not separable for each $i=1,\ldots ,d$ because the reduced density operator of $|\Omega _{i}\rangle \!\langle \Omega _{i}|$ onto any qu*d*it subsystem is not rank one.

For each $i=1,\ldots ,d$, we also note that $|\Lambda _{i}\rangle \!\langle \Lambda _{i}|$ is the only pure product state acting on the subspace spanned by $|\Lambda _{i}\rangle$ and $|\Omega _{i}\rangle$. To see this, let us consider any pure state $(c_{1}|\Lambda _{i}\rangle +c_{2}|\Omega _{i}\rangle )(c_{1}^{*}\langle \Lambda _{i}|+c_{2}^{*}\langle \Omega _{i}|)$ on the subspace spanned by $|\Lambda _{i}\rangle$ and $|\Omega _{i}\rangle$ with complex numbers $c_{1}$ and $c_{2}$ such that $|c_{1}|^{2}+|c_{2}|^{2}=1$. Due to the definitions of $|\Lambda _{i}\rangle$ and $|\Omega _{i}\rangle$ in Eq. (41), it is straightforward to show that any partial trace of $|\Lambda _{i}\rangle \!\langle \Omega _{i}|$ is the zero operator. Thus, the reduced density operator of $(c_{1}|\Lambda _{i}\rangle +c_{2}|\Omega _{i}\rangle )(c_{1}^{*}\langle \Lambda _{i}|+c_{2}^{*}\langle \Omega _{i}|)$ is

52 $$\begin{aligned} \mathrm {Tr}_{{\mathbb {S}}}[(c_{1}|\Lambda _{i}\rangle +c_{2}|\Omega _{i}\rangle )(c_{1}^{*}\langle \Lambda _{i}|+c_{2}^{*}\langle \Omega _{i}|)] =|c_{1}|^{2}\,|\mathrm {Tr}_{{\mathbb {S}}}(|\Lambda _{i}\rangle \!\langle \Lambda _{i}|)+|c_{2}|^{2}\,|\mathrm {Tr}_{{\mathbb {S}}}(|\Omega _{i}\rangle \!\langle \Omega _{i}|), \end{aligned}$$

where ${\mathbb {S}}$ is any $\alpha$-qu*d*it subsystem with $\alpha <d-1$. If $c_{2}$ is nonzero, then the reduced density operator in Eq. (52) is not rank one because $|\Omega _{i}\rangle \!\langle \Omega _{i}|$ is not separable, which implies that $(c_{1}|\Lambda _{i}\rangle +c_{2}|\Omega _{i}\rangle )(c_{1}^{*}\langle \Lambda _{i}|+c_{2}^{*}\langle \Omega _{i}|)$ is not separable. Thus, $|\Lambda _{i}\rangle \!\langle \Lambda _{i}|$ is the only pure product state acting on the subspace spanned by $|\Lambda _{i}\rangle$ and $|\Omega _{i}\rangle$.

Now, let us consider $\mathrm {SEP}_{i}(\mathscr {E})$ of the ensemble $\mathscr {E}$ in Eq. (41) for each $i=1,\ldots ,d$. From Eq. (47) and the definition of $\mathrm {SEP}_{i}(\mathscr {E})$ in Eq. (10), we have

53 $$\begin{aligned} \mathrm {SEP}_{i}(\mathscr {E}) =\{E\in \mathrm {SEP}\,|\,E \text{ acting } \text{ on } \text{ the } \text{ subspace } \text{ spanned } \text{ by } |\Lambda _{i}\rangle \text{ and } |\Omega _{i}\rangle \}. \end{aligned}$$

Because any separable state is a convex combination of pure product states and $|\Lambda _{i}\rangle \!\langle \Lambda _{i}|$ is the only pure product state acting on the subspace spanned by $|\Lambda _{i}\rangle$ and $|\Omega _{i}\rangle$, we have

54 $$\begin{aligned} \mathrm {SEP}_{i}(\mathscr {E}) =\big \{a|\Lambda _{i}\rangle \!\langle \Lambda _{i}|~\big |~a\geqslant 0\big \}. \end{aligned}$$

Moreover, a straightforward calculation leads us to

55 $$\begin{aligned} \langle \Lambda _{i}|(H-\eta _{i}\rho _{i})|\Lambda _{i}\rangle =0, \end{aligned}$$

which implies $\mathrm {Tr}[(H-\eta _{i}\rho _{i})E]=0$ for all *E* in $\mathrm {SEP}_{i}(\mathscr {E})$. Thus, we have $H-\eta _{i}\rho _{i}\in \mathrm {SEP}_{i}^{*}(\mathscr {E})$, and this shows the validity of Condition (15b) for each $i=1,\ldots ,d$. Finally, Condition (18b) naturally follows from Eqs. (51) and (55).

From Theorem 2, the minimum quantity $q_\mathrm{SEP}(\mathscr {E})$ in Eq. (14) is

56 $$\begin{aligned} \begin{array}{c} q_\mathrm{SEP}(\mathscr {E})=\mathrm {Tr}H=\sum _{i=1}^{d}\eta _{i}\mathrm {Tr}(\rho _{i}M_{i})=\frac{1}{2(d-1)}. \end{array} \end{aligned}$$

Moreover, the POVM $\{M_{i}\}_{i=0}^{d}$ with Eq. (51) is a LOCC measurement since it can be implemented by performing the same local measurement $\{|i\rangle \!\langle i|\}_{i=0}^{d-1}$ on all subsystems. Thus, Corollary 2 and Eq. (56) lead us to

57 $$\begin{aligned} \begin{array}{c} p_{\mathrm{L}}(\mathscr {E})=q_{\mathrm{SEP}}(\mathscr {E})=\frac{1}{2(d-1)}. \end{array} \end{aligned}$$

Examples 1 and 2 are special cases that $p_\mathrm{L}(\mathscr {E})=p_{\mathrm{SEP}}(\mathscr {E})$, or equivalently $p_\mathrm{L}(\mathscr {E})=q_{\mathrm{SEP}}(\mathscr {E})$. We also note that there exist separable-state ensembles with $p_{\mathrm{L}}(\mathscr {E})<q_\mathrm{SEP}(\mathscr {E})$, therefore $p_{\mathrm{L}}(\mathscr {E})<p_\mathrm{SEP}(\mathscr {E})$. A well-known example with $p_\mathrm{L}(\mathscr {E})<p_{\mathrm{SEP}}(\mathscr {E})$ is the domino state ensemble which can be perfectly discriminated by separable measurements but not by LOCC measurements^8^.

## Discussion

In this paper, we have considered the situation of unambiguously discriminating multipartite quantum states, and provided an upper bound $q_{\mathrm{SEP}}(\mathscr {E})$ for the maximum success probability of optimal local discrimination $p_{\mathrm{L}}(\mathscr {E})$. We have further established a necessary and sufficient condition for the Hermitian operator *H* to realize $q_{\mathrm{SEP}}(\mathscr {E})$. Moreover, we have provided a necessary and sufficient condition for the upper bound $q_{\mathrm{SEP}}(\mathscr {E})$ to be saturated. Finally, we have illustrated our results by examples in multidimensional multipartite quantum systems.

We remark that finding $p_{\mathrm{G}}(\mathscr {E})$ and $q_\mathrm{SEP}(\mathscr {E})$ in unambiguously discriminating separable quantum states can be useful in studying the phenomenon of *nonlocality without entanglement*(NLWE)^8^. For the optimal UD of a separable-state ensemble $\{\eta _{i},\rho _{i}\}_{i=1}^{n}$, the NLWE phenomenon occurs when $p_{\mathrm{G}}(\mathscr {E})$ cannot be realized only by LOCC, that is, $p_{\mathrm{L}}(\mathscr {E})<p_\mathrm{G}(\mathscr {E})$. Due to Corollary 2, $q_\mathrm{SEP}(\mathscr {E})<p_{\mathrm{G}}(\mathscr {E})$ means $p_\mathrm{L}(\mathscr {E})<p_{\mathrm{G}}(\mathscr {E})$, therefore the occurrence of NLWE. It is a natural future work to find good bounds on optimal local discrimination in other generalized state discrimination strategies such as an optimal discrimination with a fixed rate of inconclusive results^26–30^.

## Methods

In this section, we prove Theorem 1 by showing that

58a $$\begin{aligned}&p_{\mathrm{SEP}}(\mathscr {E})\leqslant q_{\mathrm{SEP}}(\mathscr {E}), \end{aligned}$$

58b $$\begin{aligned}&p_{\mathrm{SEP}}(\mathscr {E})\geqslant q_\mathrm{SEP}(\mathscr {E}). \end{aligned}$$

### Proof of Inequality (58a)

Let us assume that $\{M_{i}\}_{i=0}^{n}$ is a separable unambiguous measurement realizing $p_{\mathrm{SEP}}(\mathscr {E})$ and *H* is a Hermitian operator giving $q_{\mathrm{SEP}}(\mathscr {E})$. Since this assumption implies Conditions (11) and (15), we have

59 $$\begin{aligned} \mathrm {Tr}(M_{0}H)\geqslant 0,~\mathrm {Tr}[M_{i}(H-\eta _{i}\rho _{i})]\geqslant 0~\forall i=1,\ldots ,n, \end{aligned}$$

which lead us to

60 $$\begin{aligned} \begin{array}{c} p_\mathrm{SEP}(\mathscr {E})=\sum _{i=1}^{n}\eta _{i}\mathrm {Tr}(\rho _{i}M_{i}) \leqslant \sum _{i=1}^{n}\eta _{i}\mathrm {Tr}(\rho _{i}M_{i}) +\mathrm {Tr}(M_{0}H)+\sum _{i=1}^{n}\mathrm {Tr}[M_{i}(H-\eta _{i}\rho _{i})]=\mathrm {Tr}H=q_\mathrm{SEP}(\mathscr {E}), \end{array} \end{aligned}$$

where the second equality is due to $\sum _{i=0}^{n}M_{i}=\mathbbm {1}$. Therefore, Inequality (58a) holds.

### Proof of Inequality (58b)

We first prove Inequality (58b) when $p_\mathrm{SEP}(\mathscr {E})=0$. In this case, we claim that every $M\in \mathrm {SEP}_{j}(\mathscr {E})$ satisfies $\mathrm {Tr}(\rho _{j}M)=0$ for each $j=1,\ldots ,n$. To see this, suppose that there is a positive-semidefinite product operator $E\in \mathrm {SEP}_{j}(\mathscr {E})$ with $\mathrm {Tr}(\rho _{j}E)>0$. Since $\mathbbm {1}-E/\mathrm {Tr}E$ is obvious separable, the following POVM $\{M_{i}\}_{i=0}^{n}$ is a separable unambiguous measurement:

61 $$\begin{aligned} M_{0}=\mathbbm {1}-E/\mathrm {Tr}E,~~M_{j}=E/\mathrm {Tr}E,~~ M_{i}=0_{{\mathscr {H}}}~~\forall i=1,\ldots ,n~~\text{ with }~~i\ne j, \end{aligned}$$

where $0_{{\mathscr {H}}}$ is the zero operator on ${\mathscr {H}}$.

Moreover, the measurement of (61) gives

62 $$\begin{aligned} \begin{array}{c} \sum _{i=1}^{n}\eta _{i}\mathrm {Tr}(\rho _{i}M_{i})= \eta _{j}\mathrm {Tr}(\rho _{j}E)>0. \end{array} \end{aligned}$$

From Inequality (62) and the definition of $p_\mathrm{SEP}(\mathscr {E})$, we have $p_{\mathrm{SEP}}(\mathscr {E})>0$, a contradiction. Therefore, there is no positive-semidefinite product operator $E\in \mathrm {SEP}_{j}(\mathscr {E})$ with $\mathrm {Tr}(\rho _{j}E)>0$. Since every positive-semidefinite separable operator can be represented as a summation of positive-semidefinite product operators, we have

63 $$\begin{aligned} \mathrm {Tr}(\rho _{j}M)=0 \end{aligned}$$

for all $M\in \mathrm {SEP}_{j}(\mathscr {E})$. Equation (63) together with the definition of $\mathrm {SEP}_{j}^{*}(\mathscr {E})$ in Eq. (16) imply that

64 $$\begin{aligned} a\rho _{j}\in \mathrm {SEP}_{j}^{*}(\mathscr {E}) \end{aligned}$$

for any real number *a* and $j \in \left\{ 1,\ldots ,n\right\}$.

By letting $H =0_{{\mathscr {H}}}$, we trivially have

65 $$\begin{aligned} H\in \mathrm {SEP}^{*}. \end{aligned}$$

For each $i=1,\ldots ,n$, we also have

66 $$\begin{aligned} H-\eta _{i}\rho _{i}=-\eta _{i}\rho _{i}\in \mathrm {SEP}_{i}^{*}(\mathscr {E}) \end{aligned}$$

where the inclusion is from Eq. (64). Equations (65) and (66) imply that $H=0_{{\mathscr {H}}}$ satisfies Condition (15), therefore

67 $$\begin{aligned} q_{\mathrm{SEP}}(\mathscr {E}) \leqslant \mathrm {Tr}H=0, \end{aligned}$$

where the inequality is due to the definition of $q_\mathrm{SEP}(\mathscr {E})$. Thus, Inequality (67) and the assumption $p_{\mathrm{SEP}}(\mathscr {E})=0$ lead us to Inequality (58b).

Now, we prove Inequality (58b) when $p_\mathrm{SEP}(\mathscr {E})>0$.

#### Lemma 1.

If $E\in \mathrm {SEP}^{*}$ and $E\ne 0_{{\mathscr {H}}}$, then $\mathrm {Tr}E>0$, where $0_{{\mathscr {H}}}$ is the zero operator on ${\mathscr {H}}$.

#### Proof

The proof is by contradiction. We first note that $\mathrm {Tr}E=\mathrm {Tr}(\mathbbm {1}E)\geqslant 0$ because $E\in \mathrm {SEP}^{*}$ and the identity operator $\mathbbm {1}$ is obviously separable. Thus, let us suppose $\mathrm {Tr}E=0$.

For an arbitrary orthonormal product basis $\{|e_{i}\rangle \}_{i=1}^{D}$ of the multipartite Hilbert space ${\mathscr {H}}=\bigotimes _{k=1}^{m}{\mathscr {H}}_{k}$, we have

68 $$\begin{aligned} \begin{array}{c} \sum \limits _{i=1}^{D}\mathrm {Tr}(E|e_{i}\rangle \!\langle e_{i}|)=\mathrm {Tr}(E\mathbbm {1}) =\mathrm {Tr}E=0, \end{array} \end{aligned}$$

where *D* is the dimension of ${\mathscr {H}}$. From $E\in \mathrm {SEP}^{*}$ and $|e_{i}\rangle \!\langle e_{i}|\in \mathrm {SEP}$ for all $i=1,\ldots ,D$, we have

69 $$\begin{aligned} \mathrm {Tr}(E|e_{i}\rangle \!\langle e_{i}|)\geqslant 0~~\forall i=1,\ldots ,D. \end{aligned}$$

Equation (68) and Inequality (69) lead us to $\mathrm {Tr}(E|e_{i}\rangle \!\langle e_{i}|)=0$ for all $i=1,\ldots ,D$. Since the choice of $\{|e_{i}\rangle \}_{i=1}^{D}$ can be arbitrary, $\mathrm {Tr}(E|e\rangle \!\langle e|)=0$ for any product vector $|e\rangle \in {\mathscr {H}}$, therefore

70 $$\begin{aligned} \mathrm {Tr}(EF)=0~~\forall F\in \mathrm {SEP}. \end{aligned}$$

We note that $\mathrm {SEP}$ spans the set of all Hermitian operators on ${\mathscr {H}}$. To see this, we first note that the set of all positive-semidefinite operators on ${\mathscr {H}}_{k}$ spans the set of all Hermitian operators on ${\mathscr {H}}_{k}$ for each $k=1,\ldots ,m$. Moreover, every Hermitian operator *A* on ${\mathscr {H}}$ can be represented as a summation of product Hermitian operators,

71 $$\begin{aligned} \begin{array}{c} A=\sum _{l}\bigotimes _{k=1}^{m}A_{l,k}, \end{array} \end{aligned}$$

where $A_{l,k}$ is a Hermitian operator on ${\mathscr {H}}_{k}$ for each $k=1,\ldots ,m$. Therefore, Eq. (70) leads us to $\mathrm {Tr}(EF)=0$ for any Hermitian operator *F* on ${\mathscr {H}}$. This means $E=0_{{\mathscr {H}}}$, a contradiction. Thus, $\mathrm {Tr}E>0$. $\square$

Let us consider the set,

72 $$\begin{aligned} \begin{array}{c} S(\mathscr {E})=\left\{ \left( \sum \limits _{i=1}^{n}\eta _{i}\mathrm {Tr}(\rho _{i}M_{i})-p,\mathbbm {1}-\sum \limits _{i=0}^{n}M_{i}\right) \in {\mathbb {R}}\times {\mathbb {H}}\,\big |\, p>p_{\mathrm{SEP}}(\mathscr {E}),~ M_{0}\in \mathrm {SEP},~M_{i}\in \mathrm {SEP}_{i}(\mathscr {E})~\forall i=1,\ldots ,n\right\} , \end{array} \end{aligned}$$

where ${\mathbb {R}}$ is the set of all real numbers and ${\mathbb {H}}$ is the set of all Hermitian operators on the multipartite Hilbert space ${\mathscr {H}}$. We note that $S(\mathscr {E})$ is a convex set due to the convexity of $\mathrm {SEP}$ and $\mathrm {SEP}_{i}(\mathscr {E})$($i=1,\ldots ,n$) in Eqs. (2) and (10). Moreover, $S(\mathscr {E})$ does not have the origin $(0,0_{{\mathscr {H}}})$ of ${\mathbb {R}}\times {\mathbb {H}}$ otherwise there exists a separable unambiguous measurement $\{M_{i}\}_{i=0}^{n}$ with $\sum _{i=1}^{n}\eta _{i}\mathrm {Tr}(\rho _{i}M_{i})>p_\mathrm{SEP}(\mathscr {E})$, and this contradicts the optimality of $p_\mathrm{SEP}(\mathscr {E})$ in Eq. (12). We also note that the Cartesian product ${\mathbb {R}}\times {\mathbb {H}}$ can be considered as a real vector space with an inner product defined as

73 $$\begin{aligned} \langle (a,A),(b,B)\rangle =ab+\mathrm {Tr}(AB), ~~(a,A),(b,B)\in {\mathbb {R}}\times {\mathbb {H}}. \end{aligned}$$

Since $S(\mathscr {E})$ in Eq. (72) and the single-element set $\{(0,0_{{\mathscr {H}}})\}$ are disjoint convex sets, it follows from separating hyperplane theorem^31,32^ that there is $(\gamma ,\Gamma )\in {\mathbb {R}}\times {\mathbb {H}}$ such that

74a $$\begin{aligned} (\gamma ,\Gamma )\ne (0,0_{{\mathscr {H}}}), \end{aligned}$$

74b $$\begin{aligned} \langle (\gamma ,\Gamma ),(r,G)\rangle \leqslant 0~~\forall (r,G)\in S(\mathscr {E}). \end{aligned}$$

Suppose

75a $$\begin{aligned} \mathrm {Tr}\Gamma \leqslant \gamma p_{\mathrm{SEP}}(\mathscr {E}), \end{aligned}$$

75b $$\begin{aligned} \Gamma \in \mathrm {SEP}^{*}, \end{aligned}$$

75c $$\begin{aligned} \Gamma -\gamma \eta _{i}\rho _{i}\in \mathrm {SEP}_{i}^{*}(\mathscr {E})~\forall i=1,\ldots ,n, \end{aligned}$$

75d $$\begin{aligned} \gamma >0, \end{aligned}$$

where $\mathrm {SEP}^{*}$ and $\mathrm {SEP}_{i}^{*}(\mathscr {E})$ are defined in Eq. (16). From Conditions (75b), (75c), and (75d), the Hermitian operator $H=\Gamma /\gamma$ satisfies Condition (15). Thus, the definition of $q_{\mathrm{SEP}}(\mathscr {E})$ in Eq. (14) leads us to

76 $$\begin{aligned} q_{\mathrm{SEP}}(\mathscr {E})\leqslant \mathrm {Tr}H. \end{aligned}$$

Moreover, Condition (75a) and the definition of $H=\Gamma /\gamma$ imply

77 $$\begin{aligned} \mathrm {Tr}H\leqslant p_{\mathrm{SEP}}(\mathscr {E}). \end{aligned}$$

Inequalities (76) and (77) complete the proof of Inequality (58b).

The rest of this section is to prove Conditions (75a), (75b), (75c), and (75d).

#### Proof of (75a)

From Eq. (73), Inequality (74b) can be rewritten as,

78 $$\begin{aligned} \begin{array}{c} \mathrm {Tr}\Gamma -\mathrm {Tr}(M_{0}\Gamma )-\sum \limits _{i=1}^{n}\mathrm {Tr}\left[ M_{i}\left( \Gamma -\gamma \eta _{i}\rho _{i}\right) \right] \leqslant \gamma p \end{array} \end{aligned}$$

for all $p>p_{\mathrm{SEP}}(\mathscr {E})$ and all $\{M_{i}\}_{i=0}^{n}$ satisfying Condition (11). If $M_{i}=0_{{\mathscr {H}}}$ for all $i=0,1,\ldots ,n$, Inequality (78) becomes Inequality (75a) by taking the limit of *p* to $p_\mathrm{SEP}(\mathscr {E})$. $\square$

#### Proof of (75b)

For an arbitrary $M_{0}\in \mathrm {SEP}$ and $M_{i}=0$ for all $i=1,\ldots ,n$, $\{M_{i}\}_{i=0}^{n}$ clearly satisfies Condition (11). In this case, Inequality (78) becomes

79 $$\begin{aligned} \mathrm {Tr}\Gamma -\mathrm {Tr}(M_{0}\Gamma )\leqslant \gamma p_\mathrm{SEP}(\mathscr {E}) \end{aligned}$$

by taking the limit of *p* to $p_{\mathrm{SEP}}(\mathscr {E})$.

Suppose $\Gamma \notin \mathrm {SEP}^{*}$, then there exists $M\in \mathrm {SEP}$ with $\mathrm {Tr}(M\Gamma )<0$. We note that $M\in \mathrm {SEP}$ implies $tM\in \mathrm {SEP}$ for any $t>0$. Thus, $\{M_{i}\}_{i=0}^{n}$ with $M_{0}=tM$ for $t>0$ and $M_{i}=0$ for all $i=1,\ldots ,n$ also satisfies Condition (11). Now, Inequality (79) can be rewritten as,

80 $$\begin{aligned} \mathrm {Tr}\Gamma -\mathrm {Tr}(tM\Gamma )\leqslant \gamma p_\mathrm{SEP}(\mathscr {E}). \end{aligned}$$

Since Inequality (80) is true for arbitrary large $t>0$, $\gamma p_{\mathrm{SEP}}(\mathscr {E})$ can also be arbitrary large. However, this contradicts that both $\gamma$ and $p_\mathrm{SEP}(\mathscr {E})$ are finite. Thus, $\Gamma \in \mathrm {SEP}^{*}$, which completes the proof of (75b). $\square$

#### Proof of (75c)

The proof method is analogous to that of (75b). For each $j\in \{1,\ldots ,n\}$, let us consider an arbitrary $M_{j}\in \mathrm {SEP}_{j}(\mathscr {E})$ and $M_{i}=0$ for all $i=0,1,\ldots ,n$ with $i\ne j$. In this case, $\{M_{i}\}_{i=0}^{n}$ clearly satisfies Condition (11) and Inequality (78) becomes

81 $$\begin{aligned} \begin{array}{ll} \mathrm {Tr}\Gamma -\mathrm {Tr}\left[ M_{j}\left( \Gamma -\gamma \eta _{j}\rho _{j}\right) \right] \leqslant \gamma p_{\mathrm{SEP}}(\mathscr {E}) \end{array} \end{aligned}$$

by taking the limit of *p* to $p_{\mathrm{SEP}}(\mathscr {E})$.

Suppose $\Gamma -\gamma \eta _{j}\rho _{j}\notin \mathrm {SEP}_{j}^{*}(\mathscr {E})$, then there exists $M\in \mathrm {SEP}_{j}(\mathscr {E})$ with $\mathrm {Tr}[M(\Gamma -\gamma \eta _{j}\rho _{j})]<0$. We note that $M\in \mathrm {SEP}_{j}(\mathscr {E})$ implies $tM\in \mathrm {SEP}_{j}(\mathscr {E})$ for any $t>0$. Thus, $\{M_{i}\}_{i=0}^{n}$ consisting of $M_{j}=tM$ for $t>0$ and $M_{i}=0$ for all $i=0,1,\ldots ,n$ with $i\ne j$ also satisfies Condition (11). Now, Inequality (81) can be rewritten as,

82 $$\begin{aligned} \begin{array}{ll} \mathrm {Tr}\Gamma -\mathrm {Tr}\left[ tM\left( \Gamma -\gamma \eta _{j}\rho _{j}\right) \right] \leqslant \gamma p_{\mathrm{SEP}}(\mathscr {E}) \end{array} \end{aligned}$$

Since Inequality (82) is true for arbitrary large $t>0$, $\gamma p_{\mathrm{SEP}}(\mathscr {E})$ can also be arbitrary large. However, this contradicts that both $\gamma$ and $p_\mathrm{SEP}(\mathscr {E})$ are finite. Thus, $\Gamma -\gamma \eta _{j}\rho _{j}\in \mathrm {SEP}_{j}^{*}(\mathscr {E})$, which completes the proof of (75c). $\square$

#### Proof of (75d)

Suppose $\Gamma \ne 0_{{\mathscr {H}}}$. From Lemma 1 together with Inequality (75b), we have $\mathrm {Tr}\Gamma >0$. Thus, Inequality (75a) and the fact that $\mathrm {Tr}\Gamma >0$ guarantee $\gamma >0$.

Now, suppose $\Gamma =0_{{\mathscr {H}}}$. We have $\gamma \ne 0$, otherwise a contradiction to Condition (74a). The strict positivity of $\gamma$ follows from Inequality (75a) with $\mathrm {Tr}\Gamma =0$ and $p_{\mathrm{SEP}}(\mathscr {E})>0$. Thus, Inequality (75d) holds regardless of $\Gamma$. $\square$

## Data Availability

All data generated or analysed during this study are included in this published article.

## References

[1] Chitambar E, Leung D, Mančinska L, Ozols M, Winter A (2014). Everything you always wanted to know about LOCC (but were afraid to ask). Commun. Math. Phys..

[2] Horodecki R, Horodecki P, Horodecki M, Horodecki K (2009). Quantum entanglement. Rev. Mod. Phys..

[3] Chitambar E, Gour G (2019). Quantum resource theories. Rev. Mod. Phys..

[4] Chefles A (2000). Quantum state discrimination. Contemp. Phys..

[5] Barnett SM, Croke S (2009). Quantum state discrimination. Adv. Opt. Photon..

[6] Bergou JA (2010). Discrimination of quantum states. J. Mod. Opt..

[7] Bae J, Kwek L-C (2015). Quantum state discrimination and its applications. J. Phys. A: Math. Theor..

[8] Bennett CH, DiVincenzo DP, Fuchs CA, Mor T, Rains E, Shor PW, Smolin JA, Wootters WK (1999). Quantum nonlocality without entanglement. Phys. Rev. A.

[9] Peres A, Wootters WK (1991). Optimal detection of quantum information. Phys. Rev. Lett..

[10] Duan R, Feng Y, Ji Z, Ying M (2007). Distinguishing arbitrary multipartite basis unambiguously using local operations and classical communication. Phys. Rev. Lett..

[11] Chitambar E, Hsieh M-H (2013). Revisiting the optimal detection of quantum information. Phys. Rev. A.

[12] Ghosh S, Kar G, Roy A, Sen A, Sen U (2001). Distinguishability of bell states. Phys. Rev. Lett..

[13] Walgate J, Hardy L (2002). Nonlocality, asymmetry, and distinguishing bipartite states. Phys. Rev. Lett..

[14] Fan H (2004). Distinguishability and indistinguishability by local operations and classical communication. Phys. Rev. Lett..

[15] Duan R, Feng Y, Xin Y, Ying M (2009). Distinguishability of quantum states by separable operations. IEEE Trans. Inf. Theory.

[16] Chitambar E, Duan R, Hsieh M-H (2014). When do local operations and classical communication suffice for two-qubit state discrimination?. IEEE Trans. Inf. Theory.

[17] Bandyopadhyay S, Cosentino A, Johnston N, Russo V, Watrous J, Yu N (2015). Limitations on separable measurements by convex optimization. IEEE Trans. Inf. Theory.

[18] Bandyopadhyay S, Russo V (2021). Entanglement cost of discriminating noisy bell states by local operations and classical communication. Phys. Rev. A.

[19] Zhang J-H, Zhang F-L, Wang Z-X, Lai L-M, Fei S-M (2020). Discriminating bipartite mixed states by local operations. Phys. Rev. A.

[20] Ha D, Kim JS (2022). Bound on local minimum-error discrimination of bipartite quantum states. Phys. Rev. A.

[21] Ivanovic ID (1987). How to differentiate between non-orthogonal states. Phys. Lett. A.

[22] Peres A (1988). How to differentiate between non-orthogonal states. Phys. Lett. A.

[23] Dieks D (1988). Overlap and distinguishability of quantum states. Phys. Lett. A.

[24] Zhang J-H, Zhang F-L, Wang Z-X, Yang H, Fei S-M (2022). Unambiguous state discrimination with intrinsic coherence. Entropy.

[25] Eldar YC, Stojnic M, Hassibi B (2004). Optimal quantum detectors for unambiguous detection of mixed states. Phys. Rev. A.

[26] Chefles A, Barnett SM (1998). Strategies for discriminating between non-orthogonal quantum states. J. Mod. Opt..

[27] Zhang C-W, Li C-F, Guo G-C (1999). General strategies for discrimination of quantum states. Phys. Lett. A.

[28] Fiurášek J, Ježek M (2003). Optimal discrimination of mixed quantum states involving inconclusive results. Phys. Rev. A.

[29] Bagan E, Muñoz-Tapia R, Olivares-Rentería GA, Bergou JA (2012). Optimal discrimination of quantum states with a fixed rate of inconclusive outcomes. Phys. Rev. A.

[30] Herzog U (2015). Optimal measurements for the discrimination of quantum states with a fixed rate of inconclusive results. Phys. Rev. A.

[31] Boyd S, Vandenberghe L (2004). Convex optimization.

[32] When $$A$$ and $$B$$ are disjoint convex sets in a real vector space $$V$$ with an inner product $$\langle \cdot ,\cdot \rangle $$, there exist $$x\in {\mathbb{R}}$$ and $$\vec{v}\in V$$ such that $$\vec{v}\ne \vec{0}$$ and $$\langle \vec{a},\vec{v}\rangle \leqslant x\leqslant \langle \vec{b},\vec{v}\rangle $$ for all $$\vec{a}\in A$$ and all $$\vec{b}\in B$$.

